# 872. Number of Sexual Partners and Patient-Reported Outcomes among Older Adults Living With HIV

**DOI:** 10.1093/ofid/ofab466.1067

**Published:** 2021-12-04

**Authors:** Peter Mazonson, Jeff Berko, Theoren Loo, Giselle D Coelho, Erik Lowman, Peter Shalit, Frank Spinelli

**Affiliations:** 1 Mazonson & Santas, Inc., Larkspur, CA; 2 Mazonson & Santas Inc., Menlo Park, CA; 3 Cook County Health, Chicago, Illinois; 4 Midland Medical Center, Oakland Park, Florida; 5 ViiV Healthcare, Sag Harbor, NY

## Abstract

**Background:**

Many older (age 50+) adults living with HIV (OALWH) are sexually active. However, little is known about the relationship between number of sexual partners and mental health outcomes among OALWH.

**Methods:**

Data were utilized from the Aging with Dignity, Health, Optimism and Community (ADHOC) cohort, an observational study of OALWH from ten US clinics.

To measure sexual activity, participants were asked “How many sexual partners have you had in the last year?” with response options ranging from zero to “greater than five.” Loneliness was measured using the Three-item Loneliness Scale, and depression was measured using the Patient Health Questionnaire-2. Significance was determined by Kruskal-Wallis tests followed by unadjusted pairwise comparisons.

**Results:**

Of 1,027 participants, the mean (SD) age was 58.9 (6.1) and 876 (85%) were male. 312 (30%) had zero sexual partners in the past year, 308 (30%) had one partner, 197 (19%) had 2-5 partners, and 210 (20%) had >5 partners. Of the participants with one partner, 230 (75%) were married, coupled or partnered, and 78 (25%) were single, widowed, separated, or divorced (Single). Figure 1 shows that people with one partner were significantly less lonely than any other group (p< 0.01 for pairwise comparisons), and all other groups were statistically similar to each other. This pattern was also seen with depression (p< 0.01 for pairwise comparisons, Figure 2). Among subgroup of people with one sexual partner, those who were married, coupled or partnered were less lonely (4.41 vs. 5.67, p< 0.01) and less depressed (0.95 vs 1.38, p=0.02) than those who were single, widowed, separated, or divorced.

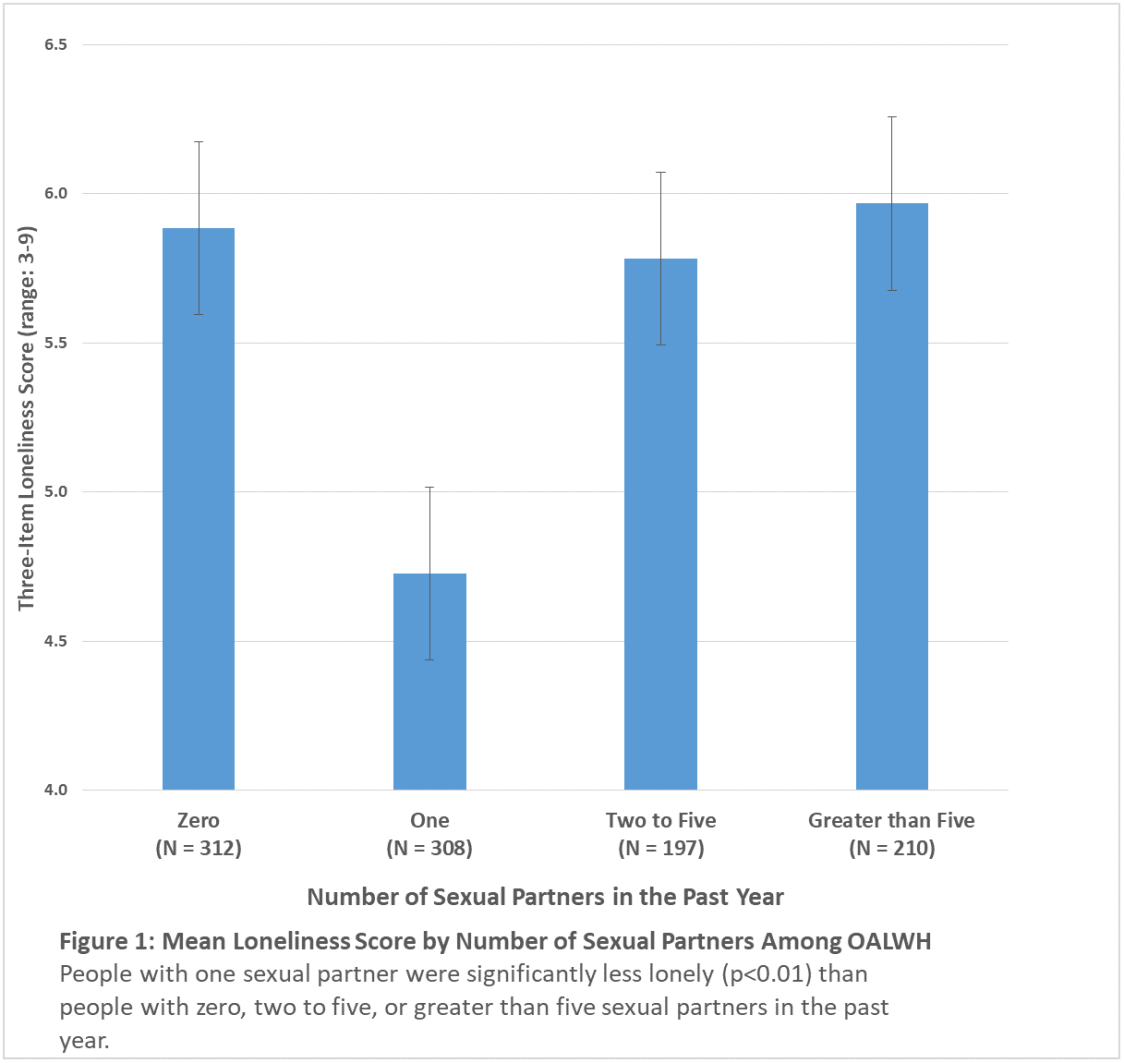

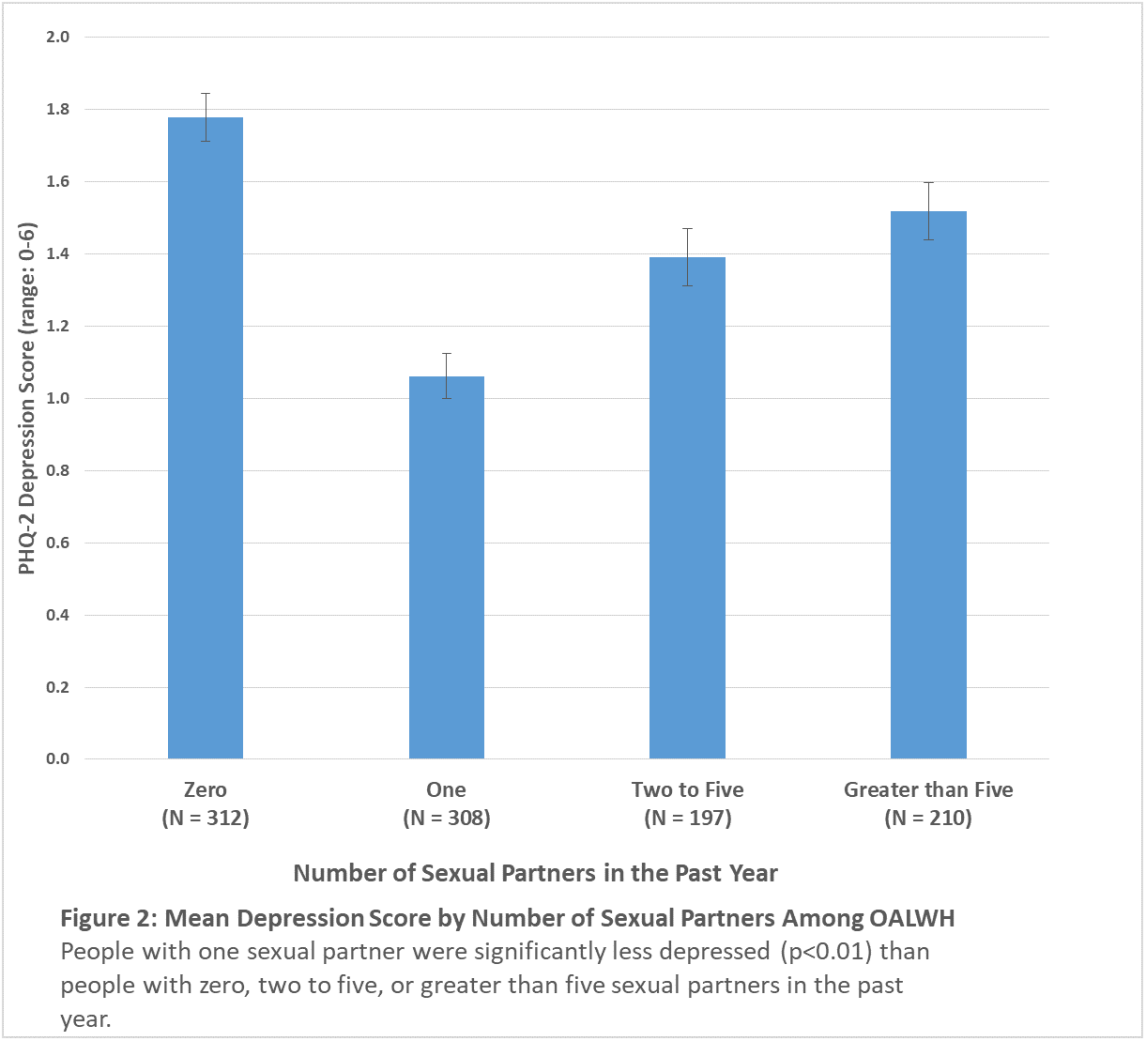

**Conclusion:**

Among OALWH, people with one sexual partner were less lonely and depressed than people with zero or with ≥2 partners. Furthermore, people with one sexual partner who were married or in a committed relationship were less lonely and depressed than people with one sexual partner who were not.

**Disclosures:**

**Peter Mazonson, MD, MBA**, **ViiV Healthcare** (Grant/Research Support) **Jeff Berko, MPH, BS**, **ViiV Healthcare** (Scientific Research Study Investigator) **Theoren Loo, MPH**, **ViiV Healthcare** (Grant/Research Support) **Giselle D. Coelho, MD. MPHTM**, **Lilly** (Research Grant or Support)**Medscape, Clinical Care Options** (Independent Contractor)**Viiv, Gilead, Janssen** (Advisor or Review Panel member, Research Grant or Support) **Erik Lowman, MD**, **Gilead** (Grant/Research Support)**Janssen** (Grant/Research Support)**ViiV** (Grant/Research Support) **Peter Shalit, MD, PhD**, **Abbvie** (Grant/Research Support)**Gilead Sciences** (Consultant, Grant/Research Support, Speaker’s Bureau)**Glaxo Smithkline** (Consultant, Grant/Research Support)**Janssen** (Consultant, Grant/Research Support, Speaker’s Bureau)**Merck** (Grant/Research Support, Speaker’s Bureau)**Thera** (Speaker’s Bureau)**ViiV Healthcare** (Speaker’s Bureau) **Frank Spinelli, MD**, **ViiV Healthcare** (Employee)

